# Higher rate of complications with uncemented compared to cemented total hip arthroplasty for displaced intracapsular hip fractures: A randomised controlled trial of 50 patients

**DOI:** 10.1007/s00590-020-02808-x

**Published:** 2020-10-17

**Authors:** N. D. Clement, Marietta van der Linden, J. F. Keating

**Affiliations:** 1 Department of Orthopaedics and Trauma, The Royal Infirmary of Edinburgh, Little France, Edinburgh, EH16 4SA UK; 2 Centre for Health Activity and Rehabilitation Research, Queen Margaret University, Edinburgh, EH21 6UU UK

**Keywords:** Total hip arthroplasty, Cemented, Uncement, Outcome, Function

## Abstract

**Background:**

The primary aim of this study was to compare the functional outcome of uncemented with cemented total hip arthroplasty (THA) for displaced intracapsular hip fractures. The secondary aims were to assess length of surgery, blood loss, complications and revision rate between the two groups.

**Methods:**

A prospective double-blind randomised control trial was conducted. Fifty patients with an intracapsular hip fracture meeting the inclusion criteria were randomised to either an uncemented (*n* = 25) or cemented (*n* = 25) THA. There were no differences (*p* > 0.45) in age, gender, health status or preinjury hip function between the groups. The Oxford hip score (OHS), Harris Hip score (HHS), EuroQol 5-dimensional (EQ5D), timed get up-and-go (TUG), pain and patient satisfaction were used to assess outcome. These were assessed at 4, 12 and 72 months after surgery, apart from the TUG which as only assessed as 6 months.

**Results:**

The study was terminated early due to the significantly (*n* = 8, *p* = 0.004) higher rate of intraoperative complications in the uncemented group: three fractures of the proximal femur and five conversions to a cemented acetabular component. There were no significant (*p* ≥ 0.09) differences in the functional measures (OHS, HSS, EQ5D, TUG and pain) or patient satisfaction between the groups. There was no difference in operative time (p = 0.75) or blood loss (*p* = 0.66) between the groups. There were two early revisions prior to 3 months post-operatively in the uncemented group and none in the cemented group, but this was not significant (log rank *p* = 0.16).

**Conclusion:**

There was a high rate of intraoperative complications, which may be due to poor bone quality in this patient group. There were no ergonomic or functional advantages demonstrated between uncemented and cemented THA. Cemented THA should remain as the preferred choice for the treatment of intracapsular hip fractures for patients that meet the criteria for this procedure.

## Introduction

Total hip arthroplasty (THA) is an accepted management option for displaced intracapsular neck of femur fractures for independent elderly patients [1]. THA relative to a hemiarthroplasty for the treatment of displaced intracapsular neck of femur fractures offers the potential of a better post-operative hip specific functional outcome and overall generic health but is associated with a higher rate of dislocation and a longer operative time [2, 3]. Cemented THA has an increased risk relative to an uncemented THA of bone cement implantation syndrome [4], which is associated with an increased early and late post-operative mortality rate [5]. Therefore uncemented THA has the potential advantage of avoiding cement which is associated with lower rates of cardiac and respiratory complications and risk of intraoperative death [5–9], and a lower post-operative mortality rate relative to cemented THA [6, 7, 10].

Data from the National Joint Registry found that 29% of all THA performed for intracapsular neck of femur fractures were uncemented, whereas 42% were cemented and the remainder were hybrid or reverse hybrid fixation [11]. Gavaskar et al. [8] found uncemented THA to be associated with a shorter operative time, lower blood loss and shorter length of stay when compared to cemented THA, however this was a non-randomised comparative study consisting of only 31 patients in each group. Dogger et al. [12] described a cohort of 115 patients undergoing an uncemented THA and concluded that uncemented THA offered comparable implant survival and complication rates to that of cemented THA. Whether there are any functional benefits of an uncemented over and a cemented THA for the management of an intracapsular neck of femur fracture is not known.

The primary aim of this study was to compare the functional outcome of patients undergoing uncemented with cemented THA for a displaced intracapsular hip fractures. The secondary aims were to assess length of surgery, blood loss, complications and revision rate between uncemented with cemented THA for displaced intracapsular hip fractures. The null hypothesis was that there was no clinically significant difference in early (12-month) functional outcome between uncemented with cemented THA for displaced intracapsular hip fractures.

## Patients and methods

### Study population

The data for this study were obtained prospectively from patients recruited to a double-blind randomised controlled trial that compared the functional outcome, activity, satisfaction, complications, blood loss, length of surgery for patients undergoing uncemented with cemented THA for displaced intracapsular hip fractures. During a 16-month period (September 2009–December 2010), 50 patients were recruited to the study, all of which presented to the study centre with their injury. Patient demographics, body mass index (BMI), ASA grade were all recorded on admission.

### Trial design

The trial was powered to detect a difference of 3.5 points in the Oxford hip score (OHS) using the reported standard deviation of 7.7 [13, 14]. A power calculation (alpha of 0.05 and beta of 0.8) estimated that 73 patients per group would be required, and in addition to allow for a dropout rate of 25% at 1 year 100 patients were to be recruited to each arm. Ethical approval was granted by the local research ethics committee. Inclusion criteria consisted of: (1) patients over the age of 60 years with a displaced intracapsular hip fracture and are admitted to the study centre and are under the care of four orthopaedic trauma surgeons, (2) patients who were independently mobile before their hip fracture, (3) patients without cognitive impairment (mini − mental score > 6) and able to give informed consent and (4) patients without serious concomitant disease. Exclusion criteria were: those not meeting the inclusion criteria, patients who are not independently mobile outside the home, unable to give informed consent and with a serious concomitant disease with anaesthetic risk too great for THA will be excluded. All patients presented to the trauma unit at the study centre and were identified by the consultant in change of the patient. Suitable patients were approached at the time of admission, approximately 24–48 h prior to surgery, and were recruited to the trial through informed consent. Once recruited, the senior surgeon allocated the patient to either an uncemented or cemented THA. This was drawn at random using sealed, numbered envelopes. The patient and researcher were blinded to the allocation and remained so throughout the trial period.

### Functional outcomes, satisfaction and pain assessment

Validated patient reported outcome measures were used to assess function pre-operatively (OHS only) and post-operative at 4, 12 and 72 months (for all measures).

The OHS consists of twelve questions assessed on a Likert scale with values from 0 to 4, a summative score is then calculated where 48 is the best possible score (least symptomatic) and 0 is the worst possible score (most symptomatic) [15].

The Harris hip score (HHS) is a combine subjective and objective assessment which contains eight items representing pain, walking function, activities of daily living, and range of motion of the hip joint [16]. The collective score ranges from 0 (maximum disability) to 100 (no disability). The index consists of subjective questions relating to pain and activities of daily living over the previous week and objective assessments of hip function and range of motion.

The EuroQoL (EQ) general health questionnaire evaluates five domains (5D,) which include: mobility, self-care, usual activities, pain/discomfort and anxiety/depression [17]. The 3L version of the EuroQoL questionnaire was used, with the responses to the five domains being recorded at three levels of severity (no, some/moderate, or unable/extreme problems). An individual patient’s health state can be reported based on a five-digit code for each domain, of which there are 243 possible health states. Each health state was converted to a single summary index by applying a weighting. These are specific to the United Kingdom (UK) population and are based on a time trade-off technique. This index is on a scale of − 0.594 to 1, where 1 represents perfect health, and negative values represent a state perceived as worse than death [18].

### Activity outcomes measured

The Timed Up and Go (TUG) test was performed 6 months post-operatively as originally described: the patient was timed while rising from an arm chair (approximate seat height 46 cm), walking at a comfortable and safe pace to a line on the floor 3 m away, turn and walk back to the chair and sit down again [19]. The patient had a practice walk before the assessment to become familiar with the test. A faster time indicates a better functional performance [19].

Subjective hip pain was assessed using a visual analogue scale from 1 to 10 and was assessed at 4, 12 and 72 months.

Patient satisfaction was assessed at 4, 12 and 72 months following surgery by asking four questions with a different focus: “Overall how satisfied are you with the results of your hip replacement surgery?” The response to each question was recorded using a five-point Likert scale: very satisfied, somewhat satisfied, neutral, somewhat dissatisfied, and very dissatisfied.

### Intraoperative measures

The length of surgery was recorded by the theatre nursing team and was defined as the time taken from the skin incision to application of the wound dressing. The blood loss was taken as an estimate according to fluids used and the overall loss at the end of the case.

### Survival

Several endpoints were defined for revision: all cause, aseptic loosening, and intension to treat (revision surgery performed or offered to patient but refused or the patient deemed too frail to undergo revision). Patient mortality data were obtained from hospital records and the Scottish Office (Communities Analytical Services, Scottish Executive Justice and Communities) to enable survival analysis to be adjusted for those patients who had died during the study period.

### Surgical procedure and implant

The surgery was performed or supervised by one of seven consultant surgeons. The surgical approach was left to the surgeon’s discretion. A posterior approach was used in seven patients and the rest utilised a direct lateral (Hardinge) approach to the hip joint. The cemented group received cemented Exeter stem with a contemporary cemented polyethylene cup (Stryker Orthopaedics, Mahwah, New Jersey, USA). The uncemented group received an uncemented Corail stem and a Pinnacle cup (Depuy, Warsaw, Indiana). A standardised rehabilitation protocol was used for all patients, with active mobilisation on the first day post-operatively.

### Statistical analysis

Data analysis were performed using Statistical Package for Social Sciences version 17.0 (SPSS Inc., Chicago, IL, USA). A Student’s t test, paired and unpaired, and a Mann–Whitney U test were used to compare scalar variables between groups. Dichotomous variables were assessed using a Chi square or Fishers exact test if less than *n* = 5 in a cell. Kaplan–Meier methodology was used to investigate implant survival [20]. A *p* value of < 0.05 was defined as significant.

### Ethics

Ethical approval was obtained for this study (REC number: 06/S0501/80) and the project was registered with the research and development department and was conducted in accordance with the Declaration of Helsinki and the guidelines for good clinical practice. The study was retrospectively registered with ClinicalTrials.gov (registration number NCT04372966).

## Results

**Fig. 1:**
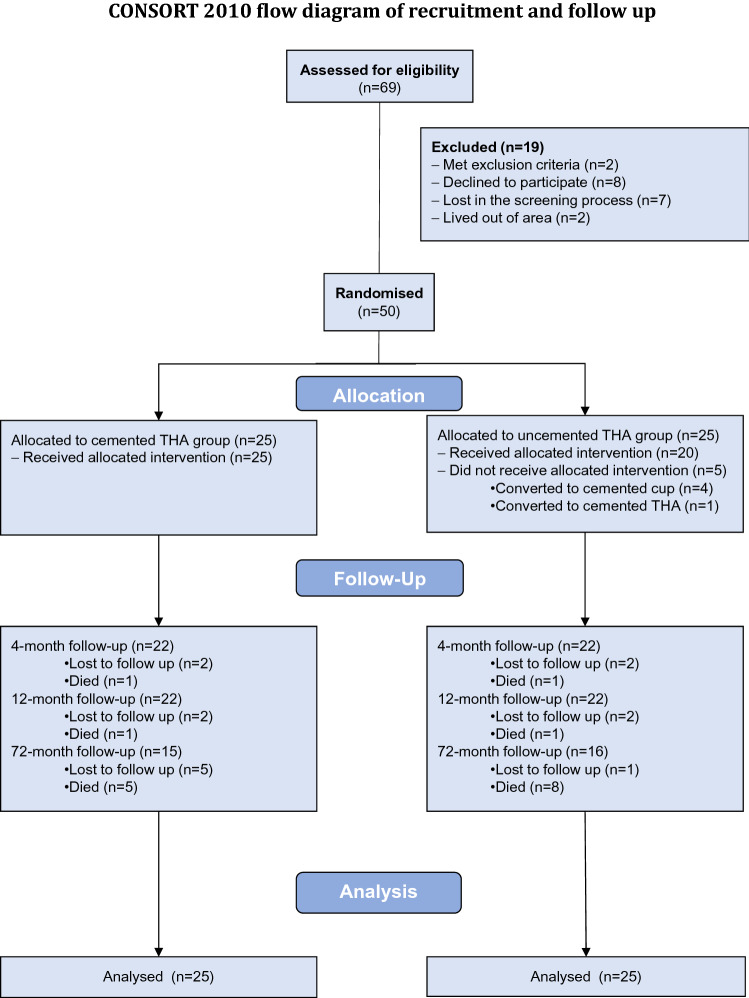
CONSORT 2010 flow diagram of recruitment and follow-up

There were 76 patients assessed for eligibility during a 16-month period (September 2009 to December 2010), of which 50 were enrolled into the study (Fig. 1). The study cohort consisted of 40 female and 10 were male patients with a mean age of 74 (range 54–87) years. There were no significant differences in gender, age, BMI, ASA grade or hip specific preoperative function between the cemented and uncemented groups (Table 1). The principal investigator (JFK) stopped enrolment to the study when only quarter of the sample size had been reached (*n* = 50), due to the fact interim analysis demonstrated the incidence of intraoperative (*n* = 8, *p* = 0.004 Fishers) and total (relative risk 11.0, 95% confidence interval 1.5–78.9, *p* = 0.002 Fishers) rate of complications were significantly higher in the uncemented group (Table 2).

**Table 1:** Patient demographics and pre-operative hip function (OHS) according to group

Demographic	Descriptive	Group		Difference/ odds ratio (95% CI)	*p*-value*
		Cemented (*n* = 25)	Uncemented (*n* = 25)		
Age (years: mean, SD)		73.3 (7.4)	74.7 (7.4)	1.4 (− 2.8–5.7)	0.94
Sex (*n*, % of group)	Male	5 (20)	5 (20)	1.0 (0.25–4.0)	0.99
	Female	20 (80)	20 (80)		
BMI (Kg/M^2^: mean, SD)		26.4 (5.5)	27.5 (7.7)	1.1 (− 1.7–2.6)	0.78
ASA (*n*, % of group)	I	3 (12)	4 (16)	N/A	0.90**
	II	20 (80)	19 (76)		
	III	2 (8)	2 (8)		
	IV	0	0		
	V	0	0		
OHS (mean, SD)		43.2 (6.5)	41.5 (9.6)	1.7 (− 2.9–6.4)	0.46

*Unpaired Students t test unless otherwise stated, **Chi-square test

**Table 2:** Significant adverse events after enrolment to the study for the first 12 months after THA

Study id	Brief description	Group	Time from surgery
002	Conversion to cemented cup	Uncemented	Intraoperative
016	Revised to cemented cup	Uncemented	2 days
017	Multiple dislocations and deep infection	Uncemented	First at 15 days
018	Conversion to cemented cup	Uncemented	Intraoperative
019	Stem subsidence	Uncemented	4 months
022	Conversion to cemented cup	Uncemented	Intraoperative
024	Conversion to cemented cup and stem	Uncemented	Intraoperative
027	Fractured femur	Uncemented	Intraoperative
042	Fractured femur	Uncemented	Intraoperative
047	Conversion to cemented cup	Uncemented	Intraoperative
048	Death—myocardial infarction	Cement	8 h
050	Fractured femur	Uncemented	Intraoperative

### Primary outcome

There was no significant difference in hip specific function according to the OHS at 4, 12 or 72 months following THA between the groups (Table 3). There was a 5-point greater (better) OHS for the cemented group at 4 months, which is clinically significant, but due to the low number of patients recruited this was not statistically significant (*p* = 0.11).

**Table 3:** Functional measures post-operatively according to group

Score (mean, SD)	Timepoint	*n*	Cemented	Uncemented	Difference (95% CI)	*p*-value
OHS	4 months	22/22	31.6 (10.9)	26.4 (8.8)	5.2 (− 1.3–11.7)	0.11
	12 months	22/24	41.0 (7.0)	40 (10.4)	1.0 (− 4.3–6.3)	0.76
	72 months	15/16	37.0 (15.4)	37.5 (12.3)	0.5 (− 11.0–12.0)	0.94
HSS	4 months	22/22	62.3 (7.1)	56.9 (10.5)	5.4 (− 1.0–11.7)	0.09
	12 months	22/24	63.6 (7.6)	65.5 (11.8)	1.9 (− 4.6–8.4)	0.55
	72 months	15/16	61.9 (11.2)	62.0 (12.0)	0.1 (− 9.1–9.2)	0.99
EQ5D	4 months	22/22	0.80 (0.21)	0.77 (0.13)	0.03 (− 0.09–0.15)	0.60
	12 months	22/24	0.85 (0.18)	0.82 (0.25)	0.03 (− 0.12–0.17)	0.72
	72 months	15/16	0.79 (0.44)	0.65 (0.43)	0.14 (− 0.27–0.55)	0.49
TUG	6 months	15/16	11.5 (4.8)	12.8 (4.9)	1.4 (− 2.2–4.9)	0.44
Pain	4 months	22/22	0.8 (1.3)	1.3 (1.5)	0.5 (− 0.5–1.5)	0.32
	12 months	22/24	0.8 (1.1)	0.8 (1.3)	0.05 (− 0.7–0.83)	0.89
	72 months	15/16	1.2 (1.2)	1.3 (1.3)	0.1 (− 0.9–1.0)	0.87

### Secondary outcomes

There was no significant difference in the HHS, EQ5D, TUG or level of pain at 4, 12 or 72 months following THA between the groups (Table 3). There was however a trend toward better scores for all the outcome measures in the cemented group relative to the uncemented group especially at the 4-month assessment timepoint. There was no difference in the rate of satisfaction at 4 (*p* = 0.71, Fishers), 12 (*p* = 0.40, Fishers) or 72 (*p* = 0.29, Fishers) months between the two groups. There was no significant difference in the Operative time or intraoperative blood loss between the groups (Table 4).

**Table 4:** Functional measures post-operatively according to group

Score	Cemented (*n* = 25)	Uncemented (*n* = 25)	Difference (95% CI)	*p*-value
Operative time (minutes, mean; SD)	72.9 (18.4)	75.0 (20.7)	2.1 (− 11.1–15.4)	0.75
Intraoperative blood loss (ml, mean; SD)	358 (188)	333 (189)	25 (− 88–137)	0.66

### Complications

There were eight intraoperative complications, of which all were in the uncemented group (Table 2). Of these three where minimally displaced proximal femoral fractures managed with cerclage wires and five were due to poor acetabula stability and were converted to a cemented socket. There were two revisions, both in uncemented group, one for early acetabula migration which was revised to a cemented socket and the other for multiple dislocations and deep infection. The patient with multiple dislocations and deep infection underwent debridement and excision arthroplasty 3 months following THA, but due to medical complications died post-operatively. A further patient with an uncemented THA had femoral stem subsidence at 4 months and was noted to have Trendelenburg gait but did not want any further surgery. In the cemented group there was a death immediately post-operatively which was thought to be due to cardiac failure, he was a 62-year-old male with a past medical history of type II diabetes with microvasculopathy and left ventricular systolic dysfunction.

### Survival

**Fig. 2:**
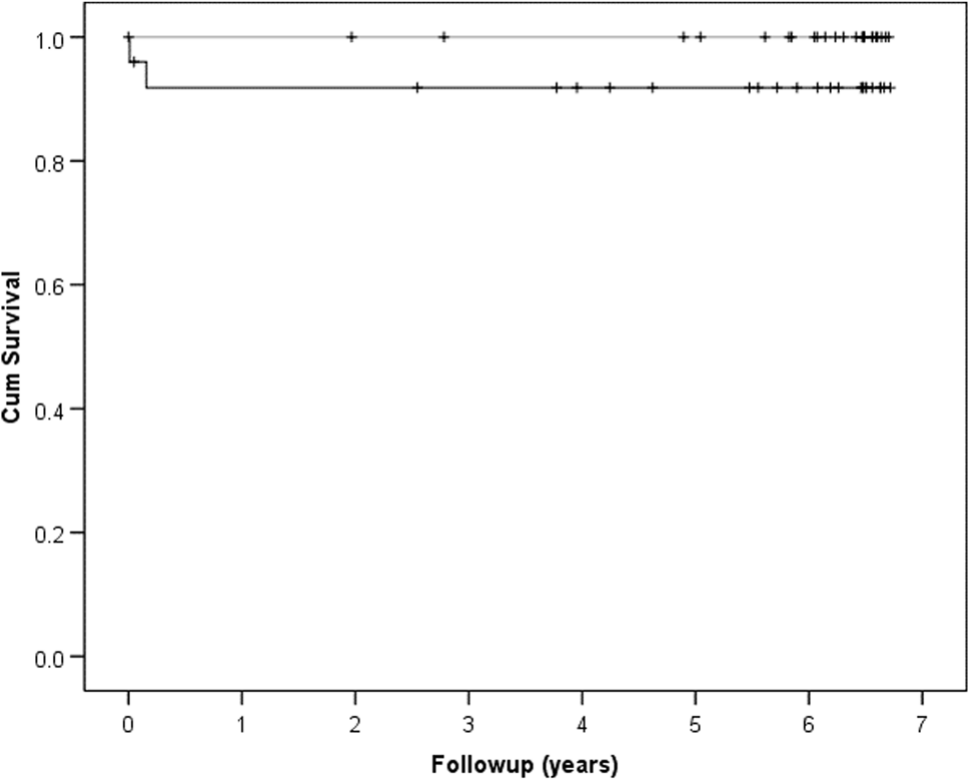
Kaplan Meier curves for the cemented (grey line) and cemented (black line) groups, for which there was no significant difference (log rank *p* = 0.16). The + signifies a censor point

Thirteen patients died during the follow-up period (median 6.2 years). There were two revisions in the uncemented group (92% survival), that were due to early complications described above, and none in the cemented group (Fig. 2), but this was not significant (log rank *p* = 0.16).

## Discussion

This randomised trial has shown the rate of complications to be higher in patients undergoing uncemented compared with cemented THA for displaced intracapsular fractured neck of femur. There were no differences in hip-specific function, overall general health or patient satisfaction between the uncemented and cemented THA groups. There was no observed benefit of the uncemented THA in operative time or blood loss when compared to the cemented THA. There were two early (less than 3 months) revisions in the uncemented THA groups and no revisions in the cemented THA group but this was not significant.

The main limitation of this study was the small number of patients assessed due to the decision to stop recruiting because of the high complication rate in the uncemented THA group. This resulted in the study being underpowered to the primary outcome measure, being the OHS, and post hoc analysis found the power to be only 35%. The strength to the current study is the design, being the only randomised controlled trial that the authors are aware of, comparing uncemented with cemented THA in patients with displaced intracapsular hip fractures. Despite being underpowered to show a hip specific functional difference between the groups this study has shown the complication rates of uncemented THA to be significantly higher than cemented THA and this may be more clinically relevant to a patient’s outcome.

The primary aim of this study was to assess whether there was any functional benefit of uncemented over cemented fixation of THA for patients with a displaced intracapsular fractured neck of femur. There was a trend towards a clinically, being greater than the minimal clinical important difference of five points [21], better OHS at 4 months in the cemented THA group but this was not statistically significant. This may be a type II error in view of the limited number of patients recruited. Liu et al. [22] compared the functional outcome of patients undergoing an uncemented with a cemented stem as part of a THA for patients with an intracapsular hip fracture. They found a significantly better hip specific function according to the HHS in those undergoing a cemented stem at a minimum of 5-year follow-up. In contrast Gavaskar et al. [8] did not find a difference in the HSS between patients undergoing an uncemented with those undergoing a cemented THA performed for hip fracture at a mean follow-up of 3 years, but their study only included 51 patients in total.

One of the potential advantages of the uncemented relative to cemented THA is a shorter operative time and lower volume of blood loss [7, 8, 23], but this was not observed in the current study. This may be due to the increased rate of intraoperative complications in the uncemented THA group, with five procedures being converted to a cemented cup and three proximal femoral fractures were incurred requiring cerclage wire fixation, which would have increased the operative time and blood loss. The 10-min shorter operative time associated with an uncemented, compared to a cemented, primary THA for osteoarthritis was not be recognised in the current study for patients with an intracapsular hip fracture [7, 23]. However, if this were the case it seems unlikely such a small difference would contribute to a significant improvement in post-operative outcomes for this patient group.

The rate of complications in the current study for uncemented THA are consistent with other authors comparing the uncemented with cemented stem fixation for THA and for hemiarthroplasty for patients with an intracapsular hip fracture [6, 24, 25]. In addition the current study has as also highlighted the difficulty of uncemented acetabula fixation with five intraoperative conversions to cemented fixation and early revision of an uncemented acetabula component because of loss of position. Chammout et al. [6] performed a randomised controlled trial of uncemented versus cemented stem fixation as part of a THA for patients with an intracapsular hip fracture. They also had to stop their trial early, with only a half of patients recruited, due to the significantly increased rate of complications in the uncemented group. Similar to the current study Chammout et al. [6] described three intraoperative femoral fractures, three dislocations and one unstable stem, which led them to conclude not to recommend uncemented femoral stems for THA in hip fracture patients.

The current study demonstrated a 92% and a 100% survival rate of the uncemented and cemented THA at 6 years, respectively. Data from the National Joint Registry demonstrated the risk of revision of uncemented relative a cemented THA for an intracapsular hip fracture was significantly increased (hazard ratio 1.8) when adjusting for confounding variables [11]. This supports the findings of the current study which also showed a lower survivorship for uncemented THA and there seems to be no survivorship benefit over a cemented THA.

## Conclusion

There was a high rate of complications in patients undergoing an uncemented compared to cemented THA for their displaced intracapsular neck of femur fracture, which may be due to poor bone quality in this patient group. There were no ergonomic or functional advantages demonstrated between uncemented and cemented THA. Cemented THA should remain as the preferred choice for the treatment of displaced intracapsular hip fractures for patients that meet the criteria for this procedure.
